# Mr. Holland's Case of Nervous Affection

**Published:** 1805-12

**Authors:** T. Holland

**Affiliations:** Sandhack, Cheshire


### *To the Editors of the Medical and Physical Journal.*

Gentlemen,

SINCE the days of Sydenham, and other more ancient authors, diseases have assumed a far different aspect; many have been imported into this island of a truly deplorable kind, while others rage with latency and obscurity, and from their insidiousness are extremely difficult to be detected.

CASE.

Mr. H. aged forty-one, of a strong, robust, athletic constitution, and of character extremely temperate, was, at the express desire of his friends, requested to consult me; not that he manifested any idea of the slightest indisposition whatever, for, to use his own expressions, relative to medical assistance, for I cannot convey them more clearly than by giving them verbatim, viz. "I feel perfectly well, am free from pain, and possess an appetite as good, nay, if not better than at any period

---

of my life, but I must confess that I have restless nights." His family were, notwithstsnding this firm and powerful assertion, seriously alarmed for his safety, his looks being ghastly and cadaverous, together with a reduction of muscular power and florid countenance, which until then he had universally assumed; at last, at their impressing solicitations, he assented to their project, and I was desired to visit him. I found him cheerful, yet his pulse vibrating 120 in the minute, irregular and intermittent, the heart palpitating to a great degree, the sound of which I could distinctly hear against the ribs, and the nervous system struggling under a most powerful degree of irritability. These unpleasant symptoms and alarming appearances, placed me in considerable difficulty relative to the plan of treatment that might be most successfully employed. I therefore hesitated not a moment in declaring to one of his friends, the unfavourable opinion I had formed, and requested a consultation might be held. A physician of eminence, philanthropy, and observation, and for whose abilities I shall ever entertain the highest respect, was twice applied to; he proposed and most strenuously recommended the tonic plan of treatment, which was partially put in execution, but not sufficiently to warrant us in the expectation we had formed of its efficacy in this extraordinary case, for, I must confess, we could neither of us give it a name.

A few doses of medicine not effecting a cure, he, for a time, relinquished them altogether, in hopes the uncertain malady would prove its own physician. But, alas! he was egregiously mistaken, he hourly grew worse; seemed fully awoke to his deplorable situation, and expressed a wish that I might be again consulted. I accordingly visited him, and found all the symptoms previously enumerated, considerably exasperated in their nature and tendency; appetite impaired, and a degree of pyrexia pervading the whole system, with a depression of the mental functions.

Suggesting to myself, that the quickness of pulse, and increased action of the vascular system, might be caused by an organic affection of the heart, or some morbid derangement of the vessels closely connected therewith, together with the great irritability, induced me to have recourse to that class of medicines which have a power of reducing the quick action of the heart, and of restoring the lost energy of the nervous system; therefore determined upon trying the digitalis and castoreum, aided by the tonic powers of chalybeates and myrrh.

Never

Never did medicine for a time more conspicuously promote its object; the pulse became regular, the heart assumed its natural functions, nervous irritability ceased, and there were strong marked appearances of the general return of former looks and good health; but this important and favourable omen soon subsided, his complaints returned with a degree of violence that wounded the feelings of all who saw him; it was then considered a hopeless case, and that he would soon arrive "at that bourne from whence no traveller returns." However, as a dernier resort, the exibition of mercury was concluded upon; the system became early influenced by its powers, its sovereign effects arrested the progress of the disease, and crowned him with the blessings of health to the great joy of his family and a large circle of friends; and I feel a pride in attesting, that a period of twelve months has elapsed since the completion of the cure, with a full enjoyment of every comfort.

I am, &c.

*Sandback, Cheshire,*

*Oct.* 21, 1805.

T. HOLLAND.

